# Cold atom microwave clock based on intracavity cooling in China space station

**DOI:** 10.1038/s41526-024-00407-2

**Published:** 2024-06-06

**Authors:** Siminda Deng, Wei Ren, Jingfeng Xiang, Jianbo Zhao, Lin Li, Di Zhang, JinYin Wan, Yanling Meng, XiaoJun Jiang, Tang Li, Liang Liu, Desheng Lü

**Affiliations:** 1 Aerospace Laser Technology and Systems Department, Shanghai Institute of Optics and Fine Mechanics, Chinese Academy of Sciences, Shanghai, 201800 China; 2 Key Laboratory of Quantum Optics, Shanghai Institute of Optics and Fine Mechanics, No. 390 Qinghe Road, Jiading District, Shanghai, 201800 China; 3 Center of Materials Science and Optoelectronics Engineering, University of Chinese Academy of Sciences, Beijing, 100049 China

**Keywords:** Atomic and molecular physics, Optical physics

## Abstract

Atomic clocks with higher frequency stability and accuracy than traditional space-borne atomic clocks are the cornerstone of long-term autonomous operation of space-time-frequency systems. We proposed a space cold atoms clock based on an intracavity cooling scheme, which captures cold atoms at the center of a microwave cavity and then executes in situ interactions between the cold atoms and microwaves. As a result of the microgravity environment in space, the cold atoms can interact with the microwaves for a longer time, which aids in realizing a high-precision atomic clock in space. This paper presents the overall design, operational characteristics, and reliability test results of the space atomic clock based on the intracavity cooling scheme designed for the operation onboard the China space station. In addition, the engineering prototype performance of the space cold atoms microwave clock is also presented. The ground test results for the clock show a fractional frequency stability of 1.1 × 10^−12^ τ^−1/2^ reaching 2.5 × 10^−15^ at 200,000 s, providing solid technical and data support for its future operation in orbit.

## Introduction

In recent years, there has been a notable increase in missions involving human space exploration activities ranging from near-Earth to deep space objectives, including lunar development^1^, Mars landing^2^, and solar marginal exploration^3,4^. It is necessary to establish a precise space-time reference frame in deep space to provide accurate navigation, positioning, and communication services for large numbers of spacecraft^5,6^. In this reference frame, atomic clocks that operate autonomously for long periods and keep time precisely will play an important role. At present, satellite navigation atomic clocks have stabilities of 1 × 10^−14^ and rely on frequent calibrations with high-performance ground-based atomic clocks^7–9^. Cold atomic clocks with higher frequency accuracy and stability, or mercury ion–trapped clocks with high-frequency stability and small size, may be potential candidates.

Several advanced space atomic clocks have demonstrated their potential for future applications in space, including the Cold Atom Clock Experiment in Space (CACES)^10–12^ and Projet d’Horloge Atomique à Refroidissement d’Atomes (PHARAO)^13–15^ clocks and the Deep Space Atomic Clock (DSAC)^16^. CACES was launched in 2016 and successfully operated onboard China’s Tiangong-2 space laboratory for over 30 months with estimated short-term frequency stability of 3 × 10^−13^ τ^−1/2^. The PHARAO project will provide an Atomic Clock Ensemble in Space (ACES) clock signal with a fractional frequency instability better than 1 × 10^−16^ and an inaccuracy in the 10^−16^ regime. The flight model of PHARAO demonstrated a stability of 3.2 × 10^−13^ τ^−1/2^, passing the qualification process. On ground, DSAC demonstrated a short-term fractional frequency stability of 1.5 × 10^−13^ τ^−1/2^, and after 12 months of operation in space, it demonstrated a long-term stability of 3 × 10^−15^ at 23 days. The CACES and PHARAO clocks use a ring waveguide microwave cavity to separate the oscillating field and excite atomic transitions. This approach produces narrower atomic spectral lines by reducing the initial velocity of the atomic cloud; this enhances the performance of the atomic clock. However, the duty cycle of the microwave interrogation within a single clock cycle decreases as the launched velocity of the atomic cloud decreases, which limits improvements in short-term stability.

In efforts to advance the application of cold atom clocks, numerous research groups have made significant strides in their miniaturization^17–24^. Ascarrunz et al.^19^ have developed a portable cold atom clock based on rubidium. Besides, Liu et al.^20^ and Esnault et al.^21^ reduced the physics package and streamlined the clock cycles by employing isotropic laser cooling. Müller et al.^22^, Lee et al.^23^, and Bregazzi et al.^24^ investigated the potential of 3D MOT intracavity cooling to augment the compactness of cold atom clocks. To further improve the performance of a space-based cold atomic clock compared with CACES and explore the potential for future miniaturization, we previously proposed a Cold Atom Microwave Clock in Space (CAMiCS) based on the intracavity cooling of rubidium-87 atoms^25^. In the intracavity cooling space atomic clock, combined with a low-velocity intense source (LVIS)^26^, the rubidium atoms are cooled, state-selected, interrogated, and then detected in the center of a cylindrical microwave cavity that resonates with the clock frequency. Compared with the ring microwave cavity scheme, the intracavity cooling scheme bypasses the time duration of the atomic cloud passing through the different functional zones from the capture zone to the state selection, Ramsey interrogation^27^, and detection zones in the vacuum chamber during each clock cycle. This design can largely reduce the dead time during the clock cycle, reducing the influence of the Dick effect^28^. Considering the Dick effect caused by the phase noise of a local oscillator, if a BVA crystal oscillator is used as the local oscillator, the frequency stability can reach 6.0 × 10^−14^ τ^−1/2^, and if an ultra-stable laser or other ultra-low phase noise local oscillator is used, the frequency stability can reach 3.8 × 10^−14^ τ^−1/2^. CAMiCS is planned for use onboard the Mengtian lab module of the China space station, combined with a hydrogen maser and an optical lattice clock, to form an atomic clock ensemble with the aim of realizing an autonomous precision time reference in space. In this paper, we report the design and ground test performance of the CAMiCS engineering prototype.

## Methods

### Integrated design of the microwave clock

**Fig. 1:**
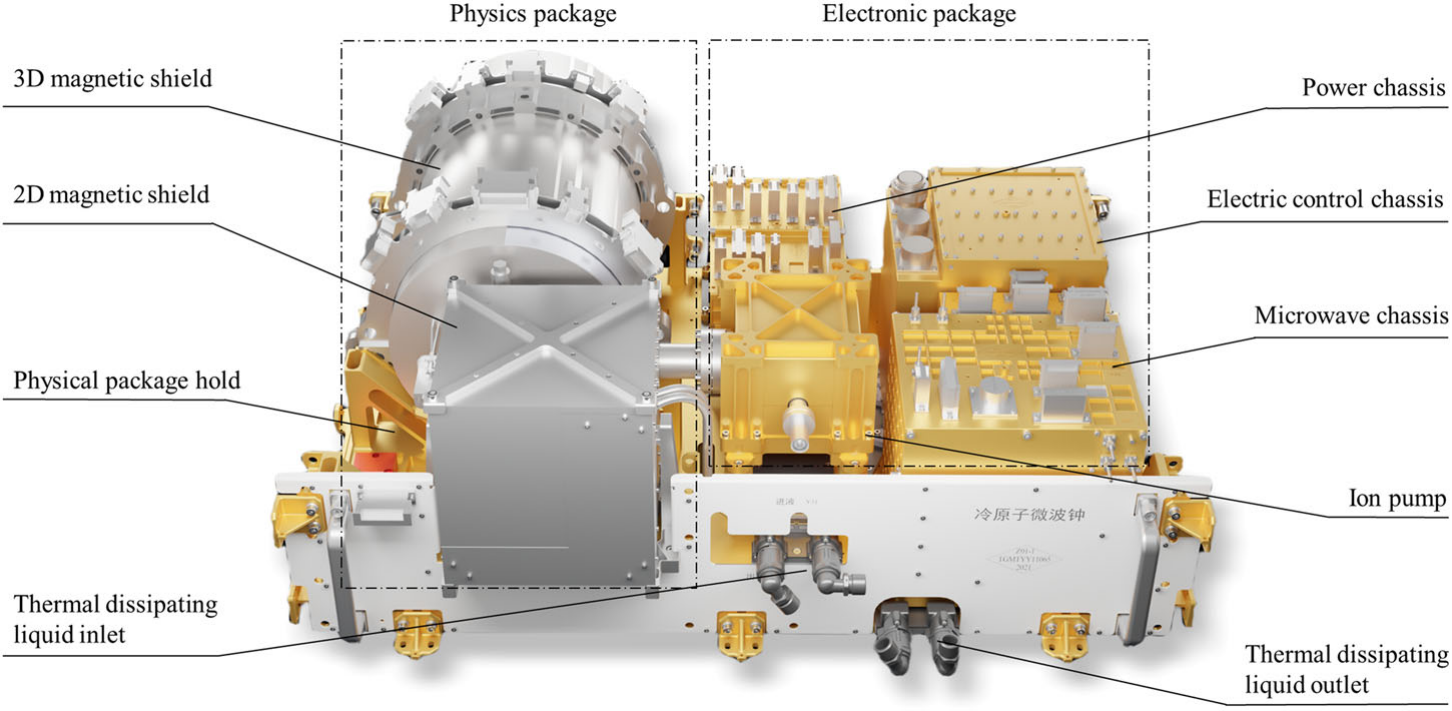
Schematic of the space cold atomic clock in the Mengtian lab module of the China space station. The physics and electronic packages are highlighted in the dashed boxes. The optical bench is located under the physics package.

CAMiCS, as a maintainable experimental cabinet, is installed in a standardized equipment rack of the Mengtian lab module in the Tiangong space station. A schematic of CAMiCS is shown in Fig. 1. The equipment rack provides cooling liquid to CAMiCS through the front panels and supplies power and communication interfaces through the back sockets. The physics package and the optical bench are located on the left side of the CAMiCS model. The optical bench is located under the physics package and, therefore, cannot be seen from the perspective shown in Fig. 1. The electronics package is on the right side of the model; here, the microwave frequency synthesizer chassis^29^ provides two 6.834-GHz microwave signals fed into the microwave cavity in the physics package, The acousto-optic chassis is designed to provide drivers for seven acousto-optic modulators (AOM) situated on the optical bench. The amplitude and frequency of these signals are adjusted separately by voltage-controlled oscillators and power amplifiers. Notably, two direct digital synthesizers (DDSs) govern the frequency of the signals on the two AOMs, regulating the frequency of the two cooling laser beams along the microwave cavity axis. The controller chassis provides timing signals for the operation and manages the status of the entire atomic clock via FPGA and DSP. The power conversion chassis converts the 100 V power supply provided by the equipment rack into 15, 12, and 5 V, as well as any other special power needs, for all of the chassis. The separate design of these electronics chassis serves to facilitate astronauts in replacing or upgrading them as necessary during the long-term operation of the space station. The heat produced by electronic components is dissipated through the equipment rack liquid cooling loop system. The temperature of some critical components, such as the microwave cavity, optical bench, and microwave chassis, are actively controlled by thermo-electric coolers (TEC). The overall size of CAMiCS is approximately 900 mm × 600 mm × 400 mm, and it weighs approximately 105 kg.

**Fig. 2:**
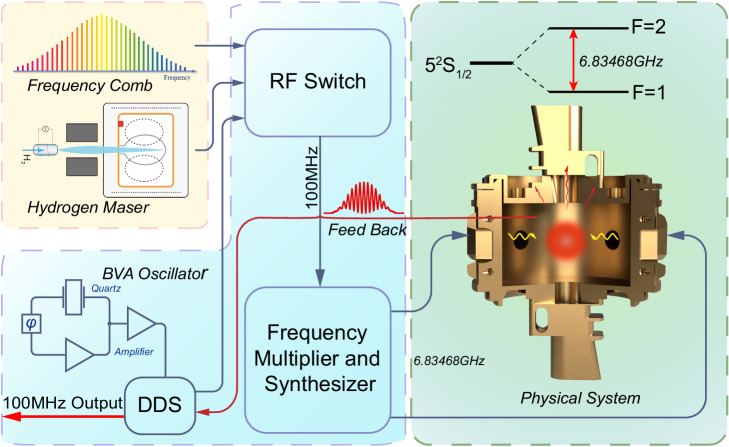
Closed-loop logic diagram of the atomic clock. The RF switch enables selecting and switching among the three 100-MHz microwave sources, which are generated separately using an optical comb, an active hydrogen maser, and DDS referenced by a BVA high-stability crystal. Using a frequency synthesizer, the 100-MHz signal is multiplied to 6.83468 GHz and then fed into the microwave cavity to interact with the prepared cold atoms. The feedback error signal is then sent to either the DDS or the ultra-stable laser after comparing the frequency with the fine energy level of the rubidium atoms to achieve a highly stable 100-MHz closed-loop output. This is achieved via frequency discrimination of the cold rubidium atoms.

There are three atomic clock signal interfaces on the front panel of CAMiCS, including one 100-MHz output and two 100-MHz inputs. The two external input 100-MHz signals are separately generated by a hydrogen maser and an optical frequency comb (OFC) on board the Mengtian lab module. The closed-loop logic of the clock is illustrated in Fig. 2. Either one of the external 100-MHz signals or the internal 5-MHz BVA oscillator can be used as the local oscillator input for the microwave frequency synthesizer chassis. The internal 5-MHz BVA oscillator generates another 100-MHz signal with the same power as the external 100-MHz signal using a frequency synthesizer; One of three signals is then selected via an RF switch. The selected 100-MHz signal is multiplied and synthesized to generate two 6.834-GHz signals, which are coupled into a microwave cavity to interrogate the prepared cold atoms. If the internal 5-MHz BVA oscillator is selected, the frequency error messages of the 6.834-GHz signals discriminated by the cold atoms are transferred from the controller chassis to the frequency synthesizer in the microwave chassis and then output as a corrected 100-MHz signal. If the hydrogen maser–generated 100-MHz signal is selected, the frequency error messages are recorded to analyze the difference between the rubidium clock and the hydrogen clock and to evaluate the frequency uncertainty of the rubidium clock under the influence of external parameters such as the magnetic field, atomic density, atomic number, microwave field, and temperature. If the frequency comb–generated 100-MHz signal is selected, the ultra-low phase noise microwave signal generated by the comb can reduce the impact of the Dick effect on the frequency stability during the microwave clock operation and obtain a more stable clock signal. In this mode, frequency error feedback is achieved via communication between the microwave clock and the ultra-stable laser.

### Design of the physics package

The physics package depicted in Fig. 1 includes a cylindrical five-layer magnetic shield that encircles the microwave cavity and a square two-layer magnetic shield that surrounds the LVIS. All of the magnetic shields are designed to minimize the effect of magnetic field fluctuations on the LVIS and the clock transition frequency during the orbital operation of the space station. The magnetic shielding coefficient was tested by simulating the magnetic environment in orbit. The results indicate that even if there is a magnetic field variation of ±40 μT^30,31^, the residual magnetic field intensity variation at the center of the microwave cavity is less than ±0.3 nT. In addition, the impact of the magnetic field variation on the second-order Zeeman frequency shift of the atomic clock is less than 1 × 10^−16^.

**Fig. 3:**
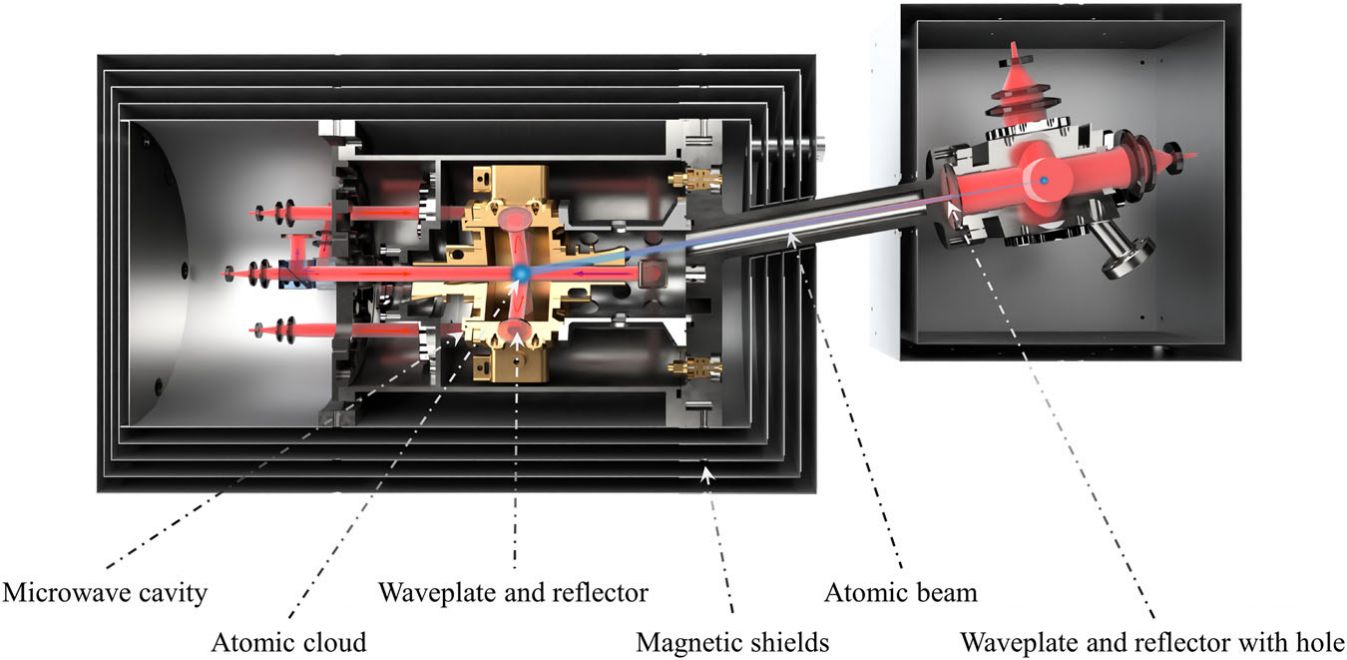
Operational schematic of the clock. Atoms are pre-loaded in a titanium cavity with the LVIS method and form an atomic beam, and the atoms in the atomic beams are leaked into the microwave cavity by a 2 mm hole on a waveplate with highly reflective coating through a differential tube. Subsequently, these atoms are cooled at the center of the cavity through the optical molasses method.

The core parts of the physics package inside the three-dimensional magnetic shield are shown in Fig. 3. The cold atoms in the beam generated by the LVIS on the right side are captured and cooled by the optical molasses^32^ in the center of the microwave cavity on the left side. In the LVIS scheme, the diameter of the three orthogonal laser beams is 25 mm, the power of the beams is greater than 20 mW, and the magnetic field gradient generated by a pair of magnetic field coils is ~5 G cm^−1^ at the center of the laser beam intersection, which can produce a cold atom beam of ~10^9^ s^−1^. The cold atomic beam generated by the LVIS passes through a differential pumping tube with a diameter of 2 mm and a length of 12.5 mm. After traveling ~160 mm, the cold atoms enter the microwave cavity through an 8-mm-diameter hole with a deviation angle of 10° from the cutoff waveguide and are captured by the optical molasses lasers formed by four laser beams coming from the optical bench. Two of the three-dimensional molasses laser beams, with a diameter of 9 mm, are introduced through two 10-mm-diameter cutoff waveguides with 90° turning orthogonally on the side wall of the microwave cavity; the resulting beams are separately reflected by the quarter-wavelength waveplates with high reflective coatings on the rear surface located opposite on the side wall. The other two beams in the cutoff waveguide direction propagate opposite, and a detection laser beam combined with one of them forms a standing wave to excite atomic fluorescence during the detection process. The design facilitates the formation of a traveling wave by turning off one of the two beams in the cutoff waveguide direction to push atoms during the detection and state-selection process.

The TE011-mode cylindrical microwave cavity is structurally adjusted to guarantee resonance with atomic transition frequency 6.834 GHz at 26 °C in vacuum, corresponding to the hyperfine level separation of the ground state of the rubidium-87 atom. During clock operation, the cavity temperature is then actively controlled within ± 0.1 °C. The opening on the wall of the microwave cavity has a certain attenuation on the Q factor of the microwave cavity. However, the test results show that the loaded Q factor of the microwave cavity is still approximately 4800. According to our simulation analysis, the current design does not affect the overall microwave field distribution in the cavity. The microwave cavity is installed in a cylindrical vacuum chamber maintained at a pressure of 5 × 10^−8^ Pa by an ion pump and getters. The outer wall of the cylinder chamber is wound with a magnetic coil, which generates a constant magnetic field of approximately 100 nT directed along the axis of the cylinder; this provides the quantized orientation for state selection and the microwave interrogation of the cold atoms.

### Design of the optical bench

**Fig. 4:**
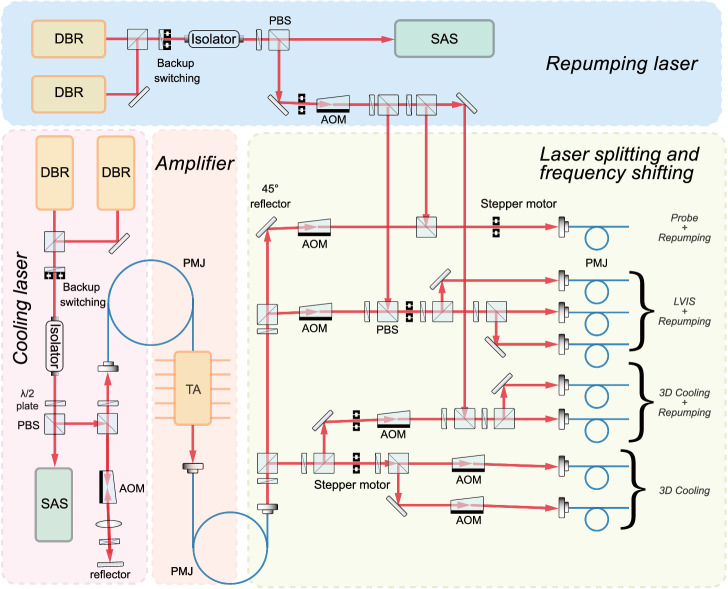
Schematic diagram of the optical bench. The cooling laser, after frequency stabilization via the saturated absorption spectra, is amplified by a tapered amplifier (TA) and then frequency-shifted by 5 AOMs according to the different needs of the apparatus, resulting in a total output of eight lasers with a total power greater than 240 mW. Of these, the repumping laser, after frequency stabilization and frequency shifting, is coupled into six other fibers.

The optical bench provides three beams of cooling lasers required for the LVIS in the physics package, four beams for the optical molasses inside the microwave cavity, and one beam for the detection laser. The generation and distribution of these laser beams are shown in Fig. 4. The laser source includes four distributed Bragg reflector (DBR) laser diodes and one tapered amplifier (TA). The DBR lasers employ a cold backup scheme to improve their reliability. Two of the DBRs work as cooling lasers, and the other two work as repumping lasers. The frequency of the cooling laser is stabilized on a crossover resonance between the F = 2 → F’ = 3 and F = 2 → F’ = 2 transitions in the saturated absorption of the ^87^Rb D_2_ line, and the frequency of the repumping is stabilized on a crossover resonance between the F = 1 → F’ = 2 and F = 1 → F’ = 1 transitions. Each DBR laser has an output power of approximately 100 mW, and the TA laser amplifier outputs a total laser power of ~400 mW from a single-mode polarization-maintained fiber. The TA output is ultimately coupled into eight single-mode polarization-maintaining fibers after an appropriate beam combination and frequency conversion. Meanwhile, the output of the repump laser is combined with one probe beam, three LVIS beams, and two optical molasses beams.

The frequency of the output laser also needs to be shifted appropriately using the acousto-optic modulator (AOM) on the optical bench to meet the requirements of laser cooling and atom detection. At the same time, the AOM is used as a fast power controller for the lasers. In addition, the laser power can be completely turned off by the mechanical optical shutters. The combination of these two methods, together with several stray light reduction measures on the optical bench, can result in a laser power turn-off ratio of more than 140 dB such that the atomic clock frequency shift influenced by the residual light in the cavity is below an order of 10^−17^ ^33^. Besides, we shifted the light frequency far from resonance during the interrogation period to further prevent the light frequency shift.

**Fig. 5:**
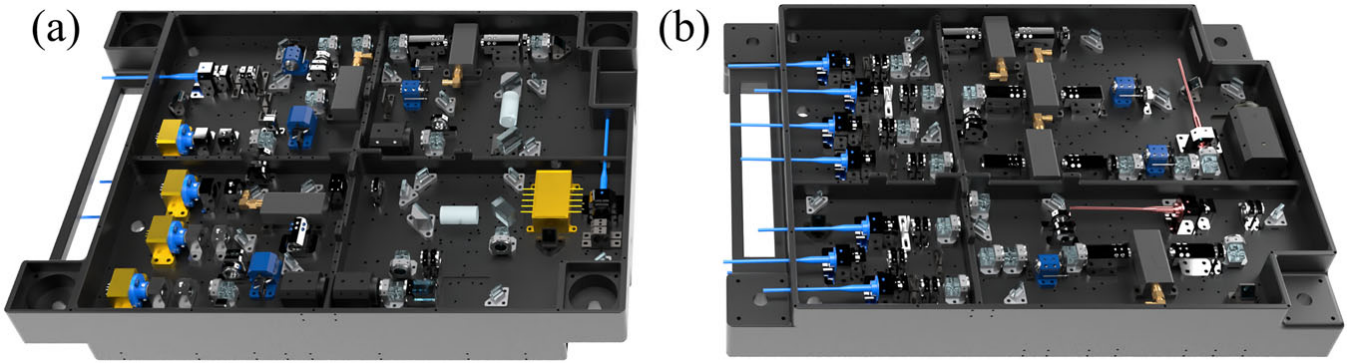
Optical bench structure diagram. **a** Laser source and amplifier side; **b** Laser splitting and frequency shifting side.

The structure diagram of optical bench is depicted in Fig. 5. The optical bench is constructed from the SiC-reinforced Al–Si alloy material and incorporates a dual-sided configuration with the size of 484 mm × 338 mm × 84 mm to enhance the space utilization rate. The miniaturized free space laser technology is inherited from CACES^34^ with the addition of designs such as tapered amplifier (TA) component^35,36^.

### Time sequence of the clock

**Fig. 6:**
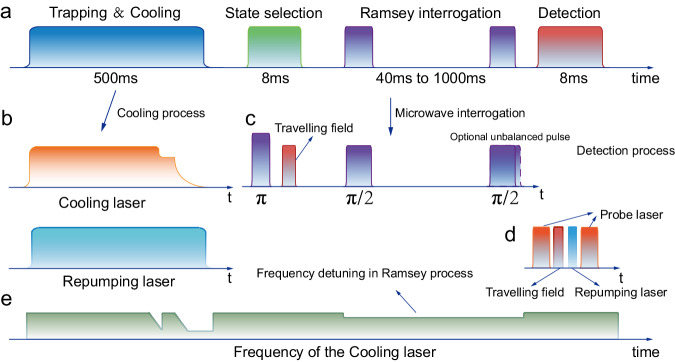
Operating time sequence of the atomic clock. **a** Complete process flow, including the trapping and cooling, state selection, Ramsey interrogation, and detection processes. **b** Specifics of the cooling phase, particularly the power trends of the cooling laser and the repumping laser. **c** Details of the microwave interrogation process, including a microwave-optical state selection process and a classic Ramsey process. Importantly, CAMiCS would correct the microwave power imbalance induced by free expansion through symmetry control of the two microwave pulses. **d** Specifics of two probe lasers, traveling wave fields, and repumping lasers in the detection process. **e** Frequency variation of the cooling laser including pre- and post-cooling and the detuning in the Ramsey process.

Figure 6 illustrates the operation sequence of CAMiCS. During the capture and cooling period of the cold atoms, the optical molasses captures ~5 × 10^7^ atoms from the cold atomic beam within 500 ms. Then, the LVIS cooling beams are turned off, and the atoms undergo a polarization gradient cooling process in the following 2 ms. During this process, the laser power and frequency are scanned to decrease the temperature of the atomic cloud to several micro-Kelvin. Once the laser beams are turned off using the mechanical optical shutters, the cold atom cloud will expand freely at the center of the microwave cavity. At the surface of the Earth, the atomic cloud is accelerated by gravity and flies out of the microwave cavity; accordingly, all subsequent atomic clock steps need to be completed before the cloud leaves the microwave cavity. However, there is no such limitation in a space microgravity environment.

During the process of atomic cloud expansion, the first step is to select cold atoms using a π microwave pulse and a traveling laser beam pulse over a period of 8 ms. The remaining rubidium-87 atoms are prepared at the ground state hyperfine level |F = 1, m_F_ = 0 >. Then, the atoms are excited to the superposition states of |F = 1, m_F_ = 0> and |F = 2, m_F_ = 0> using two π/2 Ramsey microwave pulses. Finally, the transition probability of the atoms between the |F = 2, m_F_ = 0> and |F = 1, m_F_ = 0> states is obtained by the double-level detection method^37^ in which the atomic fluorescence signals at the two energy levels are detected separately with the timing sequence in Fig. 6d. The frequency difference between the excitation microwave frequency and the rubidium clock transition frequency is processed to provide an adjustment amount for the microwave frequency. The output 100-MHz frequency reference can be adjusted appropriately with the help of the feedback loop in Fig. 2.

The interval time between the two π/2 Ramsey microwave pulses is inversely proportional to the Ramsey linewidth. Taking a longer interval time will lead to a narrower Ramsey linewidth; it is possible under these conditions that the atomic clock will have better frequency stability and accuracy. Under the ground-level operation of the microwave clock, because of the limited time that the cold atoms are in the microwave cavity, the time interval can be maintained at most for tens of milliseconds. However, in microgravity environments, cold atoms can remain in the microwave cavity for a long time. During the free expansion process of the atomic cloud in the microwave cavity, the number of cold atoms will decay exponentially with time as a result of thermal expansion and background atomic collisions, resulting in a decrease in the detection signal-to-noise ratio (SNR). Therefore, we need to comprehensively consider the linewidth and the SNR to select the optimal parameters for clock operation.

### Reporting summary

Further information on research design is available in the Nature Research Reporting Summary linked to this article.

## Results and discussion

In this section, we present the ground-based test results of the engineering prototype of CAMiCS; these tests include the atomic clock performances and several space qualification tests.

Following the integration of all of the components of CAMiCS, several critical parameters, such as the Ramsey linewidth, optimal microwave power at the π/2 transition, and center frequency of the Ramsey fringes, need to be determined before the clock can operate continuously. During the ground tests, CAMiCS was positioned such that the microwave cavity was aligned parallel to gravity to ensure the atoms’ trajectory along the microwave cavity’s axis. To extend the Ramsey interrogation time, the atom cloud underwent polarization gradient cooling and then was launched to a height of ~12 cm by adjusting the frequency detuning of the two independent cooling laser beams. During ground testing, in a single clock cycle, the time window was ~100 ms from the start of launching the atomic cloud to the end of detection. Within this time frame, state selection, Ramsey interrogation, and double-level detection were performed, considering the start and stop times of the mechanical and optical shutters. The maximum Ramsey interrogation time possible within this 100 ms was 40 ms.

**Fig. 7:**
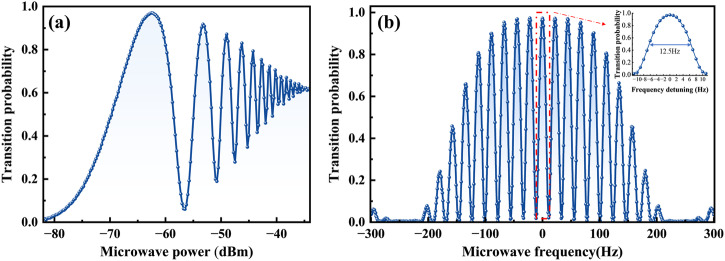
Atomic population oscillations after microwave interrogation. **a** Transition probability versus microwave power, and **b** Ramsey fringe with a 40-ms free evolution time.

Figure 7a shows the Rabi oscillation, a well-known phenomenon that arises from the interaction between atoms and a single microwave pulse. The Rabi curve demonstrates the process of scanning the microwave power to identify the power point of the π transition when the microwave frequency is fixed at the center frequency of the atomic clock transition with only one interaction between the atom and the microwave. The first transition peak in the figure represents the optimal microwave power of the π transition, and the transition probability is close to unity, indicating that all atoms in the cloud sense the uniformity of the microwave field in the cavity. The Ramsey fringes, shown in Fig. 7b, depict the change in the transition probability obtained by scanning the microwave frequency under the interaction of two π/2 microwave pulses. The central frequency of the fringes represents the target point of microwave frequency locking. With a Ramsey interrogation time of 40 ms, the half-width of the central peak is ~12.5 Hz.

After conducting the Rabi oscillation and Ramsey fringe tests, the entire microwave clock underwent rigorous space qualification tests to assess its suitability for the rocket launch process. The tests comprised mechanical vibration and thermal cycling to evaluate the clock’s robustness under harsh environmental conditions. During the mechanical test, the clock was subjected to 90 s of 3 g rms of random vibration ranging from 20 Hz to 2000 Hz. The thermal cycling test included 12.5 cycles spanning the temperature range from 10 to 35 °C with the change rate of 1 °C min^−1^. Following the environmental tests, we reassessed the laser power, frequency, quantity of captured atoms, and both Rabi and Ramsey fringes under same conditions. The findings indicate no significant alterations in the critical parameters of CAMiCS before and after undergoing mechanical and thermal cycling tests, demonstrating the clock’s resilience and viability for space missions.

**Fig. 8:**
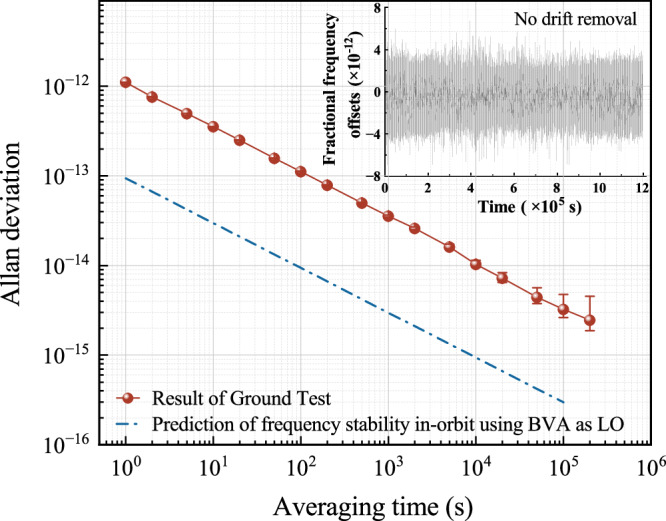
Frequency stability of the proposed device compared with hydrogen clocks and in-orbit stability prediction. The red points and line indicate the stability of the atomic clock, which reaches 1.034 × 10^−14^ at 10,000 s. The blue dashed lines represent the frequency stability prediction in orbit.

During the closed-loop operation stage, the internal BVA oscillator on CAMiCS serves as the local oscillator (LO). The microwave interrogation frequency is precisely modulated at the half-width of the Ramsey fringe. Subsequently, the adjustment value of the interrogation microwave frequency is determined by the fluctuation of the transition probability and the slope of the central fringe after each clock cycle. This calculated adjustment is then acted on the DDS to maintain the 6.834-GHz output of the frequency synthesizer precisely at the center of the Ramsey fringes. To assess the output 100-MHz frequency stability, a comparison was made with a 100-MHz signal from a T4 science hydrogen maser in the laboratory. The fractional frequency stability, depicted in Fig. 8, exhibits an Allan deviation of approximately 1.1 × 10^−12^ τ^−1/2^, which reaches 2.5 × 10^−15^ at a specific time of 200,000 s. The frequency stability constraints analysis of CAMiCS on ground reveals that the short-term stability is predominantly limited by the Dick effect and technical noise in the detection process. During the orbital operation, CAMiCS extends the interaction time between atoms and microwaves, which will effectively enhance the microwave duty cycle, mitigating the influence of the Dick effect. Moreover, employing a reference source with reduced phase noise contributes to reducing this effect. Meanwhile, the SNR of the detection remains relatively constant by switching the preamplifiers with different amplification factors, provided the number of detected atoms exceeds 8 × 10^4^. Consequently, the performance of CAMiCS is expected to be significantly enhanced when deployed in a microgravity environment.

**Fig. 9:**
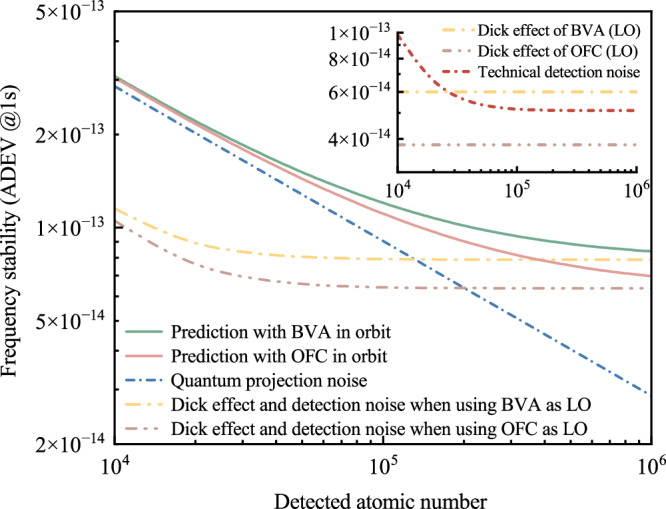
In-orbit performance analysis of CAMiCS. The green and red curves depict short-term stability when utilizing the BVA crystal oscillator and OFC as local oscillators, respectively. The blue (short dotted) line represents the quantum projection noise limit, while the yellow (dotted) line and brown (double dotted) line illustrate the combined effects of the Dick effect and detection noise for different local oscillators. The insets provide detailed illustrations of the specific influence of technical detection noise and the Dick effect.

The analysis of the short-term stability noise of CAMiCS, operating with a 0.5 Hz linewidth in-orbit, is presented in Fig. 9. To clarify the stability under different local oscillator working modes more effectively, yellow (dotted) and brown (double dotted) lines are employed to represent the cumulative effects of the Dick effect and detecting technique noise on stability when BVA and OFC serve as local oscillators respectively. The specific impacts are depicted in the insets. The findings suggest that the technical detection noise is basically independent of the atom number when such a number is higher than 8 × 10^4^, there is no notable deterioration in the SNR of technical detection noise. However, if the count of detected atoms drops below 1 × 10^5^, the short-term stability of CAMiCS is primarily constrained by quantum projection noise. Nevertheless, as the quantity of detected atoms increases, the primary noise source shifts to a combination of the Dick effect and technical noise arising from the detection process. Ground test results indicate that at an atom temperature of 5 μK and a linewidth of 0.5 Hz, the number of detected atoms is expected to diminish to 3 × 10^5^, using a Gaussian model for the cloud thermal expansion. This corresponds to short-term stability using different local oscillators, 9.4 × 10^–14^ (BVA) and 8.2 × 10^–14^ (OFC), respectively. Prior experimental results^10,12^ indicate that both the trapping efficiency and atomic temperature of cold atoms in orbit will be optimized compared to the ground results. Notably, a reduction in atomic temperature to 3 μK can lead to a twofold increase in detected atom count. Consequently, it is anticipated that CAMiCS has the potential to achieve stability better than 8.5 × 10^–14^ τ^−1/2^ and 7.1 × 10^–14^ τ^−1/2^ with different local oscillators in orbit.

This study proposed a scheme for the realization of laser cooling and the in situ detection of atoms in the center of a microwave cavity for a space clock. The proposed intracavity cooling atomic clock has the potential to achieve enhanced frequency stability in microgravity environments in comparison with traditional atomic clocks that employ atomic clouds traversing through microwave cavities and interacting with a microwave field. In addition, the proposed scheme reduces the necessary time and distance for the atoms to pass through the different functional zones of the atomic clock during each clock cycle. The focus of this study was on the engineering realization of a space atomic clock and the test results obtained on ground. The CAMiCS engineering prototype successfully passed the mechanical vibration and thermal cycle qualification tests. The prototype demonstrated an approximate frequency stability of 1.1 × 10^−12^ τ^−1/2^, with long-term stability reaching 2.5 × 10^−15^ at 200,000 s.

The analysis indicated that this type of atomic clock can achieve better performances in microgravity environments by prolonging the Ramsey interaction time. Future research on intracavity cooling should focus both on miniaturization and lightweight, as well as identifying constraints on long-term performance through uncertainty evaluation, which can provide better candidates for space-based high-precision time–frequency references.

### Supplementary information

Reporting Summary

## Data Availability

The data that support the findings of this study are available from the corresponding author upon reasonable request.

## Code availability

The code that support the findings of this study are available from the corresponding author upon reasonable request.

## References

[1] Li C (2019). Chang’E-4 initial spectroscopic identification of lunar far-side mantle-derived materials. Nature.

[2] Grotzinger JP (2012). Mars science laboratory mission and science investigation. Space Sci. Rev..

[3] Doming V, Fleck B (1995). The SOHO mission: an overview. Sol. Phys..

[4] Alonso I (2022). Cold atoms in space: community workshop summary and proposed road-map. EPJ Quantum Technol..

[5] Feng D, Guo H, Wang X, Yuan X (2014). Autonomous orbit determination and its error analysis for deep space using X-ray pulsar. Aerosp. Sci. Technol..

[6] McGrew WF (2018). Atomic clock performance enabling geodesy below the centimetre level. Nature.

[7] Senior KL, Ray JR, Beard RL (2008). Characterization of periodic variations in the GPS satellite clocks. GPS Solut..

[8] Batori E, Almat N, Affolderbach C, Mileti G (2021). GNSS-grade space atomic frequency standards: Current status and ongoing developments. Adv. Space Res..

[9] Camparo, J. Applied atomic timekeeping in space. In *2023 IEEE/ION Position, Location and Navigation Symposium* (*PLANS*) 295–310 (IEEE, 2023).

[10] Liu L (2018). In-orbit operation of an atomic clock based on laser-cooled ^87^Rb atoms. Nat. Commun..

[11] Ren W (2020). Development of a space cold atom clock. Natl Sci. Rev..

[12] Lü DS (2023). Characterization of laser cooling in microgravity via long-term operations in TianGong-2 space lab. Natl Sci. Rev..

[13] Simon E (1997). The pharao project: towards a space clock using cold cs atoms. Acta Astronaut..

[14] Cacciapuoti L, Salomon CH (2009). Space clocks and fundamental tests: the ACES experiment. Eur. Phys. J. Spec. Top..

[15] Meynadier F, Delva P, Le Poncin-Lafitte C, Guerlin C, Wolf P (2018). Atomic clock ensemble in space (ACES) data analysis. Class. Quantum Grav..

[16] Burt EA (2021). Demonstration of a trapped-ion atomic clock in space. Nature.

[17] Langlois M (2018). Compact cold-atom clock for onboard timebase: tests in reduced gravity. Phys. Rev. Appl..

[18] Esnault FX, Rossetto N, Holleville D, Delporte J, Dimarcq N (2011). HORACE: a compact cold atom clock for Galileo. Adv. Space Res..

[19] Ascarrunz, F. G. et al. A portable cold ^87^Rb atomic clock with frequency instability at one day in the 10^−15^ range. In *2018 IEEE International Frequency Control Symposium (IFCS)* 1–3 (IEEE, 2018).

[20] Liu P (2015). Scheme for a compact cold-atom clock based on diffuse laser cooling in a cylindrical cavity. Phys. Rev. A.

[21] Esnault FX, Holleville D, Rossetto N, Guerandel S, Dimarcq N (2010). High-stability compact atomic clock based on isotropic laser cooling. Phys. Rev. A.

[22] Müller ST, Magalhães DV, Alves RF, Bagnato VS (2011). Compact frequency standard based on an intracavity sample of cold cesium atoms. J. Opt. Soc. Am. B.

[23] Lee S (2021). A compact cold-atom clock based on a loop-gap cavity. Appl. Phys. Lett..

[24] Bregazzi A (2024). A cold-atom Ramsey clock with a low volume physics package. Sci. Rep..

[25] Lü, D. S. et al. Design of a space atomic clock with intracavity cooling. In *2017 Joint Conference of the European Frequency and Time Forum and IEEE International Frequency Control Symposium (EFTF/IFC)* 623–624 (IEEE, 2017).

[26] Lu ZT (1996). Low-velocity intense source of atoms from a magneto-optical trap. Phys. Rev. Lett..

[27] Ramsey NF (1958). Molecular beam resonances in oscillatory fields of nonuniform amplitudes and phases. Phys. Rev..

[28] Santarelli G (1998). Frequency stability degradation of an oscillator slaved to a periodically interrogated atomic resonator. IEEE Trans. Ultrason. Ferroelect., Freq. Contr..

[29] Li T (2018). Space qualified microwave source for cold atom clock operating in orbit. Rev. Sci. Instrum..

[30] Morić I, De Graeve C-M, Grosjean O, Laurent P (2014). Hysteresis prediction inside magnetic shields and application. Rev. Sci. Instrum..

[31] Moric I (2014). Magnetic shielding of the cold atom space clock PHARAO. Acta Astronaut.

[32] Weiss DS, Riis E, Shevy Y, Ungar PJ, Chu S (1989). Optical molasses and multilevel atoms: experiment. J. Opt. Soc. Am. B.

[33] Costanzo GA, Micalizio S, Godone A, Camparo JC, Levi F (2016). ac Stark shift measurements of the clock transition in cold Cs atoms: Scalar and tensor light shifts of the D_2_ transition. Phys. Rev. A.

[34] Ren W (2016). Highly reliable optical system for a rubidium space cold atom clock. Appl. Opt..

[35] Zhang Z (2023). Integrated, reliable laser system for an ^87^Rb cold atom fountain clock. Chin. Phys. B.

[36] Zhang Z (2022). Design of a highly reliable and low-cost optical bench for laser cooling. Opt. Fiber Technol..

[37] Bize S (1999). High-accuracy measurement of the ^87^Rb ground-state hyperfine splitting in an atomic fountain. Europhys. Lett..

